# Configurable thermoacoustic streaming by laser-induced temperature gradients

**DOI:** 10.1103/physrevapplied.23.024043

**Published:** 2025-02-18

**Authors:** Franziska Martens, Wei Qiu, Ola Jakobsson, Christian Cierpka, Andreas Ehn, Per Augustsson

**Affiliations:** 1 Department of Biomedical Engineering, Lund University, 223 63 Lund, Sweden; 2 Department of Combustion Physics, Lund University, 221 00 Lund, Sweden; 3 Engineering Thermodynamics, Technische Universität Ilmenau, 98693 Ilmenau, Germany

## Abstract

mControlling the streaming flow in acoustically actuated microchannels enables the targeted motion of suspended micro-objects. This can offer novel approaches for rare cell studies or cell sorting in medicine or basic biology. In this work, we utilize the temperature dependence of the acoustic body force, which originates from the interaction of an acoustic field with gradients in compressibility or density. A temperature gradient was optically induced inside an acoustofluidic microchannel by the absorption of light and the resulting streaming flow was measured by particle tracking in three dimensions. Inside a microfluidic channel, two different thermal fields were investigated for a fixed sound field, both in experiments and in simulations. The results show that shifting the location of the heat source from the center to the side of the channel leads to a transition from four streaming rolls to two rolls in the plane normal to the laser incidence. By modulating the optical absorbance of the medium, the streaming velocity can be tuned such that higher absorption leads to faster thermoacoustic streaming. Further, for higher absorbance, we observe increasing velocity components in the direction of the laser due to asymmetric heat generation along the beam.

## I Gradients In Acoustofluidics

Acoustofluidics, where fluids and suspended objects are manipulated by ultrasound, has become a versatile tool to separate and control cells [1–9]. In bulk-wave-acoustofluidic devices, a microchannel is actuated at its resonance frequency, typically with a piezoelectric transducer, creating a sound-pressure field in form of a standing wave [10]. The presence of standing-wave fields in a microchannel results in acoustic focusing of particles and acoustic streaming in the liquid [11]. The acoustic streaming in a standing-wave field is a net flow that typically arises from the dissipation of acoustic energy in the boundary layer if the smallest dimension of the channel geometry is comparable to the wavelength.

When density and compressibility gradients are present in a sound field, an acoustic body force arises, which can significantly impact the acoustic streaming. Suppression as well as enhancement of the acoustic streaming have been observed as effects of medium inhomogeneities in fluids with molecular gradients [12]. Given these premises, we can understand that altering the density and compressibility of a liquid can change the acoustic force and consequently the streaming pattern and velocity. One way to impose gradients in density and compressibility is by applying a spatially dependent thermal gradient.

As has been shown previously in experiments and simulations [13–15], optical heating can lead to the appearance of thermoacoustic streaming, which dominates over the boundary-driven acoustic streaming. Using a light-emitting diode (LED) and a corresponding absorbing dye dissolved in the liquid, the streaming velocity increased 50 times compared to the common boundary-driven Rayleigh streaming [16]. The LED illuminated a section extending to several hundred micrometers, corresponding approximately to the full width of the channel. The effect of a more powerful and confined heat source, creating a steeper gradient, represents a current research gap and opens the path to increased control of the resulting flow. Furthermore, the positioning of the heat source relative to the acoustic field and chamber walls, as well as the effect of varying the absorption of the light-absorbing medium, has not been investigated in previous works.

In this work, we investigate numerically and experimentally the effects of a thermal gradient, induced by focused laser light, on the acoustic streaming patterns inside an acoustofluidic resonator. That entails guiding a 50-µm-sized laser beam into a 375-µm-wide microchannel configuration to realize the heat source, while maintaining the imaging quality of a fluorescence microscope around it to allow particle tracking in three dimensions. To achieve variations in the spatial distribution of the thermal gradient, the incidence position of the laser light is altered by shifting the position of the laser from the center of the channel to a sidewall (case I) and by varying the concentration of the light-absorbing dye in order to change the absorbed fraction of the incoming light in the fluid (case II). We have found that the different positions of the heat source alter the shape of the thermoacoustic streaming. Furthermore, we show that a higher dye concentration leads to more light being absorbed in the liquid, resulting in faster thermoacoustic streaming.

## II Approach And Setup

### A Working principle

To achieve thermoacoustic streaming, we make use of the interplay between a light-induced temperature gradient and a standing-wave field. Focused laser light causes a local temperature increase as a result of light absorption in solute dye molecules. This gradient in temperature leads to gradients in density and compressibility in the fluid such that the acoustic field exerts acoustic body forces in the fluid. These body forces drive thermoacoustic streaming together with the steady boundary-driven acoustic streaming [13].

#### 1 Laser absorption in the fluid

The absorption of light in a liquid with solute molecules of concentration *c* follows the Beer-Lambert law of absorption [17–21],

(1) $$I=I_{0}e^{\alpha z},\;\;\text{with}\;\alpha =-kc,$$

(2) $$Q=-\frac{dI}{dz}=I_{0}kce^{-kcz}=\frac{2P_{\text{laser }}}{\pi r_{\text{laser }}^{2}}kce^{-kcz}.$$

The variable *k* is a constant, specific to the absorbing molecule, here indocyanine green (ICG). We measure the transmitted intensity (*I*), the incident intensity (*I*_0_), the incident power (*P*_laser_), and the spot radius (*r*_laser_). We are interested in the absorbed power per volume (*Q*). Equation (2) indicates that the thermal field created by the absorbed laser light depends exponentially on *k*. Thus, increasing the concentration of the absorbing dye molecules can be expected to lead not only to increased heat but also to an altered thermal profile. A low concentration leads to nearly uniform heating along *z*, whereas a high concentration causes the heat to be absorbed near the entry point of the light into the fluid.

#### 2 Thermal transport

Antagonistic to the laser heat source, the silicon walls of the channel, as well as the glass lid and bottom, transport the generated heat away from the fluid into the chip structure. Furthermore, the resulting temperature field in the fluid is affected by heat conduction and advection within the fluid and the surrounding solids. For a feature of characteristic length *L*, the characteristic advection time is *t*_adv_ = *L/u*_adv_ and the characteristic diffusion time is *t*_diff_ = *L*^2^*/*2*α*, for fluid velocity *u*_adv_ and thermal diffusivity of the medium *α* = *k*_th_/(*ρc*_*p*_), for thermal conductivity *k*_th_(*T*), density *ρ*(*T*), and specific heat capacity *c*_*p*_ (*T*) [22]. Assuming *L* = 375 µm, which corresponds to the channel width, and *α* ≈ 0.143 mm^2^ s^−1^ as the thermal diffusivity of water, the thermal diffusion across the channel width completes in *t*_diff_ ≈ 0.5 s. For *u*_adv_ < 380 µm s^−1^, the Péclet number Pe = *t*_diff_*/t*_adv_ = *L* · *u*_adv_*/α <* 1, indicating that the heat transfer in this case is dominated by thermal diffusion.

#### 3 Acoustic body force

Thermoacoustic streaming is driven by an acoustic body force ***f***_ac_ due to temperature-dependent gradients in compressibility *κ* and density *ρ* of the fluid [13,23]:

(3) $$f_{\text{ac}}=-\frac{1}{4}{\left|p_{1}\right|}^{2}\nabla \kappa_{0}-\frac{1}{4}{\left|v_{1}\right|}^{2}\nabla \rho_{0}$$

(4) $$=-\frac{1}{4}\left[{\left|p_{1}\right|}^{2}{\left(\frac{\partial \kappa_{s}}{\partial T}\right)}_{T0}+{\left|v_{1}\right|}^{2}{\left(\frac{\partial \rho }{\partial T}\right)}_{T0}\right]\nabla T_{0}.$$

In this work, we expect the acoustic field to be a half-wavelength standing wave across the channel. The pressure can be expressed as *p*_1_ = *p*_*A*_ sin *(k*_*y*_ *y)* and the velocity as ***v***_1_ = *v*_*A*_ cos (*k*_*y*_ *y*)**e**_*y*_, with pressure amplitude *p*_*A*_, velocity amplitude *v*_*A*_, wave number *k*_*y*_, and unit vector in the channel width (*y*) direction **e**_*y*_. The time-harmonic oscillation is implicitly assumed. In water, the first term of Eq. (3), related to the gradient in compressibility (Δ*κ*_0_), is dominant over the second term with the gradient in density (Δ*ρ*_0_). This is because the relative changes in density are small compared to the changes in compressibility for temperatures ranging from 20 to 50°C [13,24]. For simplicity, the second term is neglected in the following description of the mechanism, although both terms have been included in all calculations.

**Fig. 1:**
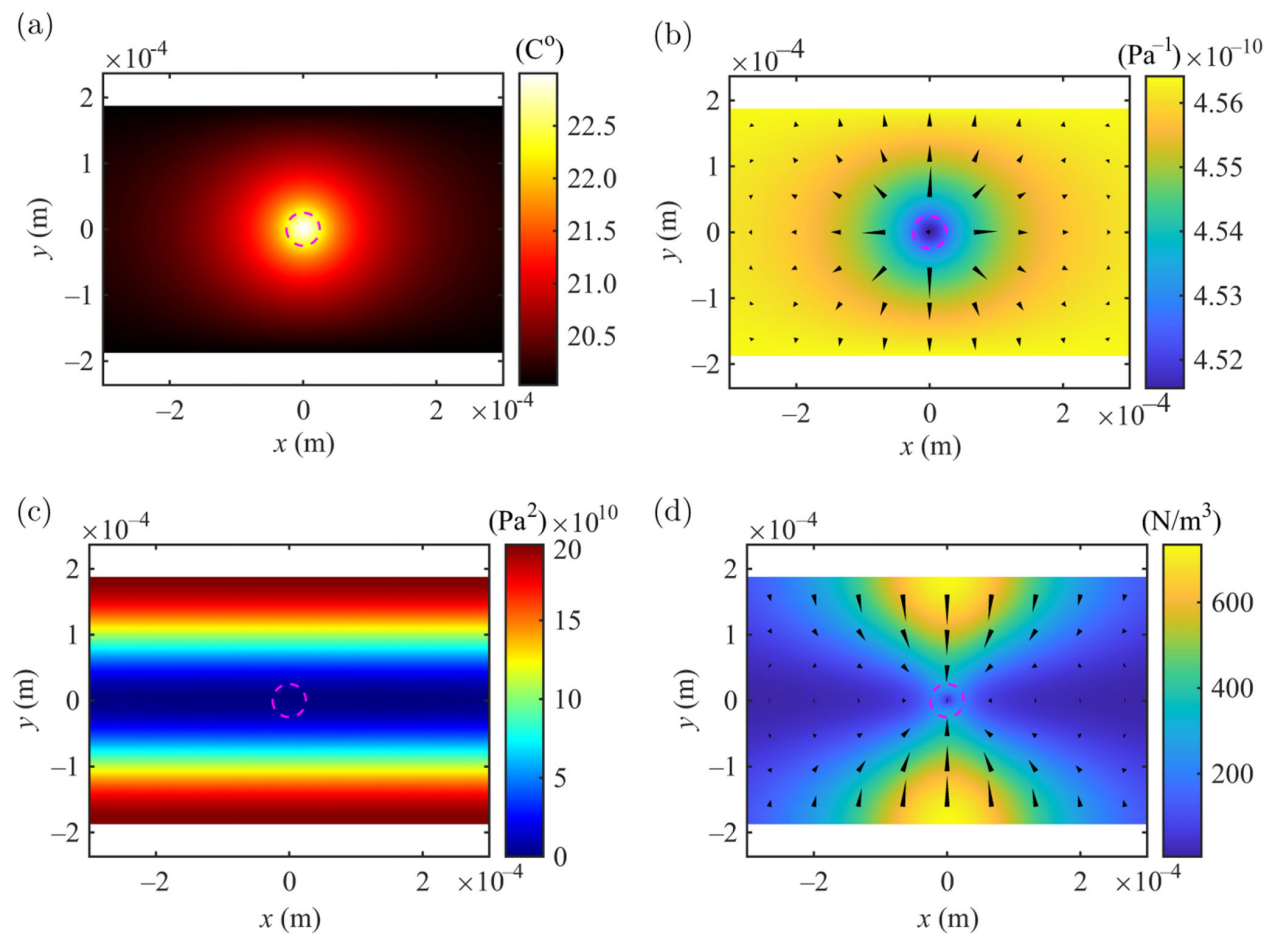
An illustration of the thermoacoustic body force. (a) A temperature field induced by the absorption of the laser light. (b) The corresponding compressibility field, with arrows indicating the direction of the gradients. (c) The acoustic pressure | *p*|^2^ field. (d) The resulting acoustic-body-force magnitude, with arrows indicating the *x* and *y* components. The dashed magenta circles indicate the 1*/e*^2^ width of the laser beam.

The presence of a thermal gradient [see Fig. 1(a)] leads to gradients in the compressibility of the fluid in the opposite direction to the thermal gradient [see Fig. 1(b)]. The body force will be directed toward the location of the temperature maximum. Factoring in the squared absolute pressure |*p*_1_|^2^ with maxima at the walls and a minimum along the channel center *y* = 0 [see Fig. 1(c)], leads to strong body forces directed inward from the walls and weak retaining forces near the channel center [see Fig. 1(d)]. This spatial asymmetry of the body force leads 024043-2 to a steady inward flow from cold regions of high-pressure amplitude, while the hot fluid tends to escape along the acoustic pressure minima. For moderate acoustic fields and small temperature gradients, the temperature field itself will not be significantly affected by the advection of the streaming flow because of the high thermal diffusivity (see Sec. II A 2). Thus, at low *p*_*A*_, the thermoacoustic streaming velocity is proportional to $p_{A}^{2}$; while for high *p*_*A*_, thermoacoustic mixing is expected to flatten the temperature gradient such that the thermoacoustic streaming velocity deviates from the linear trend. At very high acoustic fields, thermoacoustic streaming due to temperature gradients caused by friction inside the viscous boundary layer may become significant [25].

### B Setup and methods

**Fig. 2:**
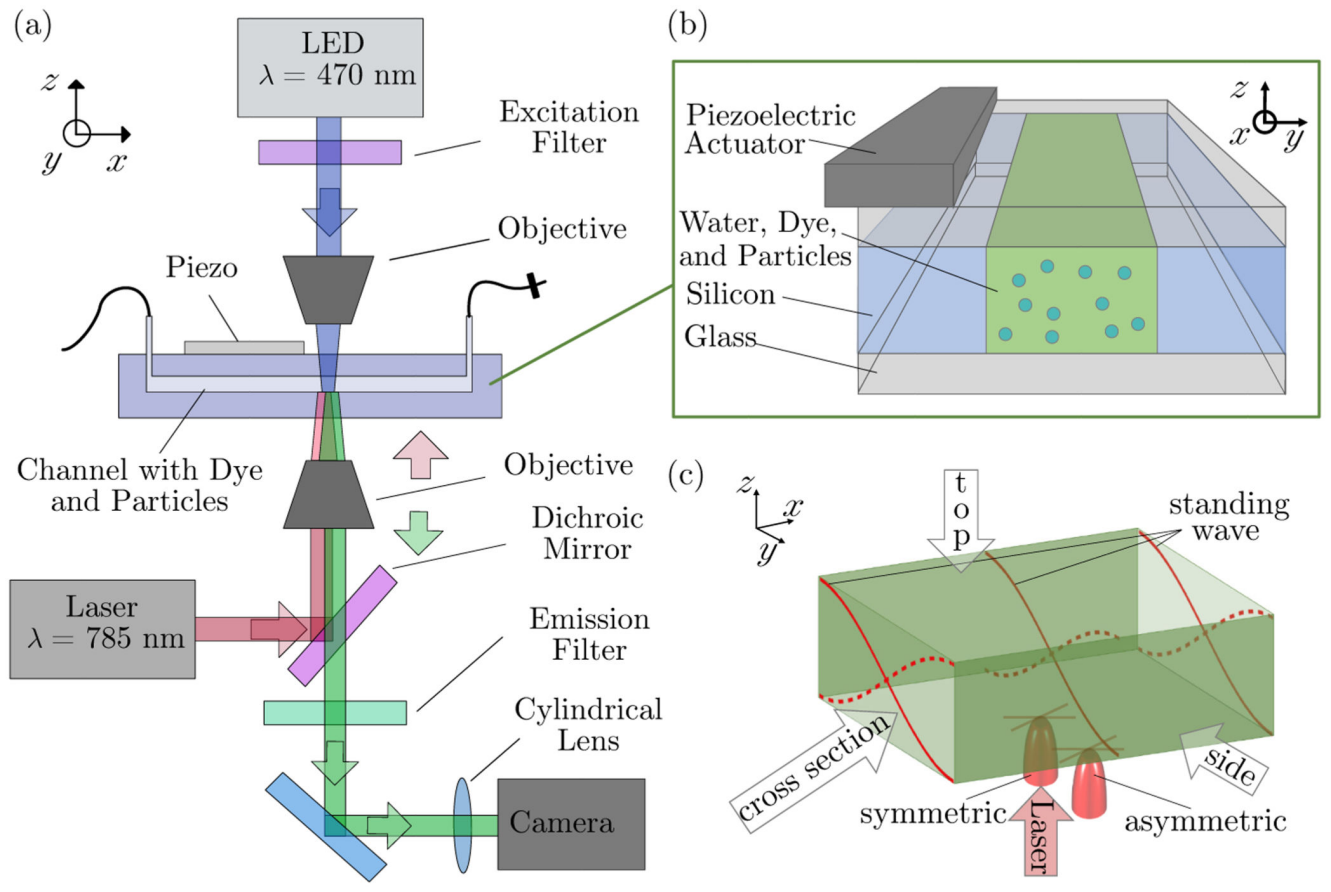
(a) The setup with laser light (785 nm) as a heat source from below and a fluorescence microscope as an imaging implement. The piezoelectric transducer generates the sound field in the microchannel with a pressure node in the center and the maxima at the walls. The thermoacoustic streaming is analyzed by defocused particle tracking through a cylindrical lens. (b) The chip consists of two glass plates and a silicon layer in which the channel is etched. A piezoelectric actuator is glued to the top glass slide, allowing light to pass through the channel. The microchannel is filled with solute molecules of an absorbing dye and 1.9-µm-sized tracer particles. (c) For experimental case I, the position of the laser was varied across the width of the channel, resulting in symmetric and asymmetric heating.

A schematic of the setup is shown in Fig. 2. In this study, the thermal field is varied in two ways: (1) the *y* position of the laser can be changed by sliding the channel sideways; and (2) the amount of absorbing dye can be varied, which influences the amount of absorbed heat and the penetration depth of light in the *z* direction (channel height). These two approaches are examined in experimental cases I and II, respectively.

#### 1 Sample preparation

The ICG dye powder (Indocyanine Green, Thermo Fisher, IR-125, laser grade, CAT 412545000) was diluted in ultrapure deionized water to the desired concentration (Table I) and spiked with 1.9-µm-diameter fluorescent tracer particles (Thermo Fisher, Fluoro-Max Dyed Green Aqueous Fluorescent Particles). These particles are large enough to provide a high signal-to-noise ratio and small enough to reliably follow the streaming flow without being affected by the acoustic radiation force. Dissolved ICG dye is known to degrade over time [26]. Therefore, measurements were conducted on the same day the samples were prepared. For case I, a transmission of Tr = 1% was chosen, corresponding to a total absorption of 99% of the incoming light.

**Fig. 3:**
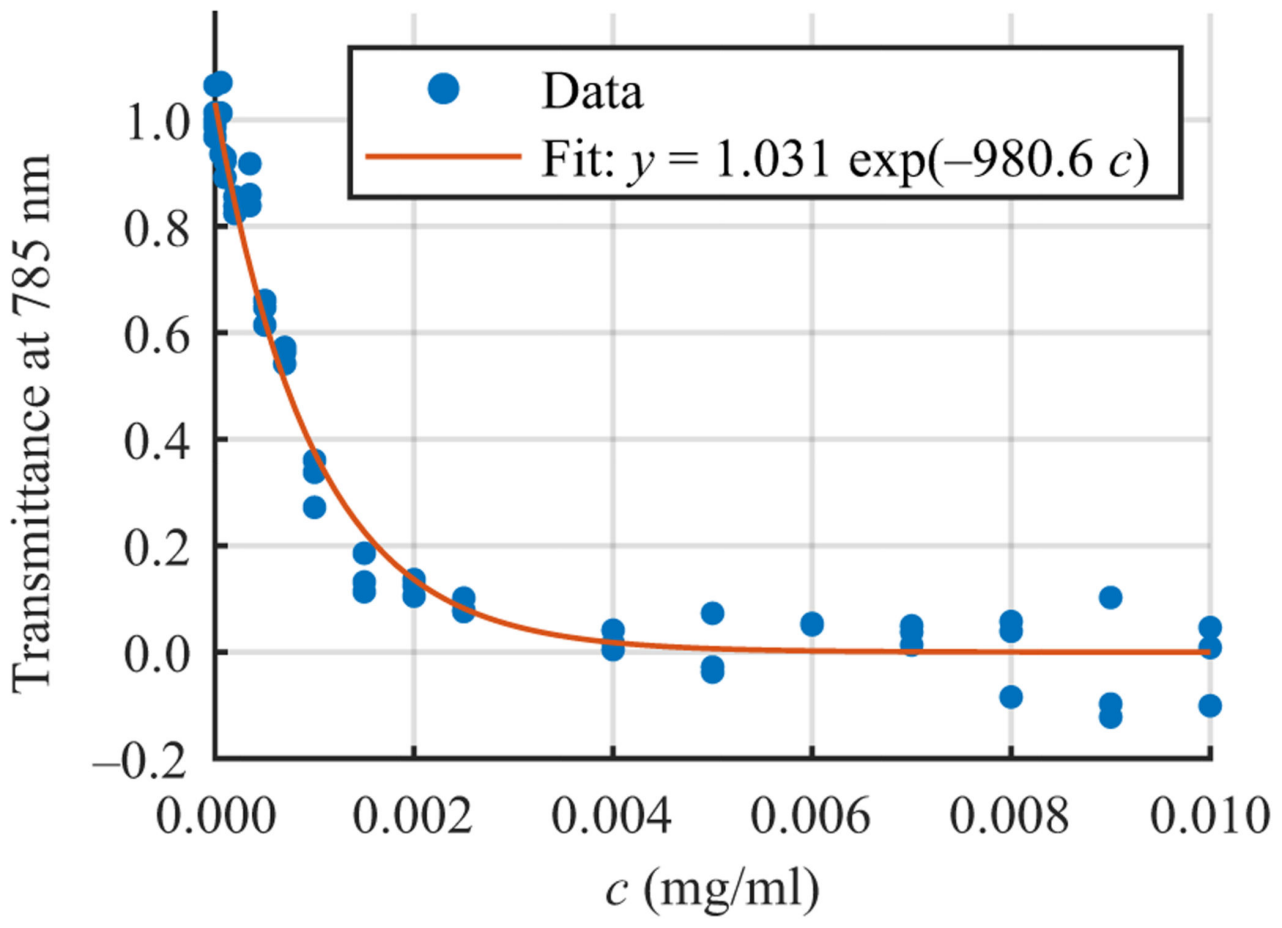
The transmission of laser light through the microchannel versus the ICG dye concentration.

To determine the relation between the absorption and the ICG concentration *c*, we measured the intensity of the transmitted light *I* through the channel for 17 concentrations of ICG using an optical power meter (Thorlabs, PM100D & S245C), as shown in Fig. 3. Following Eq. (1), we expressed the transmission through a channel of constant height as Tr = *I/I*_0_ = *A* exp (−*Bc*) and obtained *A* and *B* by fitting. By rearrangement, the concentration for a desired transmittance can be expressed as *c* = − ln (>Tr*/A*)/*B*. In Table I, we show the selected 024043-3 concentrations for studying the effect of varying fractions of absorbed light (case II).

#### 2 Description of the chip

The straight single-inlet–single-outlet microchannel (GeSim Bioinstruments and Microfluidics GmbH, Germany) has a cavity size of 375 µm in width, 150 µm in height, and 5 cm in length. The top, middle, and bottom layers of the chips are made of anodically bonded glass-silicon-glass layers, with thicknesses of 760 µm, 150 µm, and 500 µm, respectively. The thinner glass layer has two holes for inlets and outlets. The channel is etched through the silicon layer between the glass plates via deep reactiveion etching. For these experiments, the silicon walls of 024043-4 the channel function as cooling elements because of their high heat capacity. The hard materials that make up the chip allow for a standing-wave field to build up in the water-filled channel.

#### 3 Fluid transport to the chip

The inlet and outlet of the chip are connected to Teflon tubes and a Luer connector joins the tubing to a syringe with the sample liquid in it. Injection of the sample liquid is done manually and all experiments are done in stop-flow. The outlet tube is connected to a stop valve to facilitate the stop-flow [Fig. 2(a)].

#### 4 Electronics and sound

The sound field is generated with a piezoelectric transducer (Pz26, Ferroperm piezoceramics, Meggit A/S, Denmark, 30 × 4.0 × 1.0 mm) that is glued (using Loctite Super Glue, Henkel Norden AB, Stockholm, Sweden) to the glass lid of the chip [Fig. 2(b)]. The transducer is actuated by a function generator. For each of the two experimental cases, the frequency was tuned to around 2 MHz by visual evaluation of the velocity and the pattern of the acoustic streaming when no temperature gradient was present. A frequency sweep around the central frequency was used to obtain a standing-wave field invariant along *x*, through which the function generator looped over a specific sweep time (cf. Sec. II B 7 for the settings of each experiment). The transducer was driven with the function-generator amplitude set to 3*V*_pp_.

#### 5 Optics

The setup [Fig. 2(a)] comprises a 785-nm laser light source (near-infrared, continuous-wave, Toptica, IBEAM-SMART-785-S) that is guided through the microchannel to be absorbed by the dye molecules in the liquid. The laser power for all experiments was set to 10 mW, resulting in a measured power of 5.8 mW as measured through a water-filled channel. The decrease is mainly attributed to the losses in the objective. The laser is reflected onto the channel using a dichroic mirror (Thorlabs, hot mirror M254H45) and is focused using a 10× objective (Olympus UPlanFLN, 10×). At the point of incidence, the Gaussian laser spot is focused to a 1*/e*^2^ diameter of 50 µm, which is about an eighth of the channel width. The diameter of the laser spot was determined via the knife-edge method. The knife edge was mounted at the position in the sample at which the laser and microchannel met. The laser power was measured in increments of 5 µm and the result was fitted with a Gaussian curve. The 1*/e*^2^ intensity maximum was evaluated to be 50 µm in width. The chip is mounted on the manually movable stage of the microscope, which allows the chip to move with respect to the laser point of incidence in the *x* and *y* directions (for orientation, see Fig. 2). The fluorescent tracer particles are excited using an LED (Thorlabs, SOLIS LED, 470 nm, and filter FESH0500), shining through the channel from the opposite side of the laser. Before reaching the camera (CMOS, Hamamatsu, OrcaFusion BT), the light passes the dichroic mirror and an emission filter (Chroma, ET519/26m, 25 mm) and a plano-convex cylindrical lens of focal distance 300 mm (Thorlabs, LJ1558RM), positioned approximately 50 mm from the camera sensor, to 024043-5 facilitate defocus tracking (cf. Sec. II B 6). The tracer particles were imaged at a frame rate of 20 fps and an exposure time of 35 ms. The total camera field of view depicts about 1.5 × 1.5 mm^2^ of the channel. To measure the total transmission of laser light through the channel, a power meter (Thorlabs, PM100D, sensor S425C) is placed in the light path just after the laser light has passed through the channel [Fig. 2(c)].

#### 6 Flow-field analysis

Flow fields were acquired using a defocusing-based three-dimensional (3D) particle-tracking method [27–29]. Defocused microscopic images of particles at different depth positions are stored in a reference library to map the *z* component of the trajectory for each particle by cross-correlation of the particle images obtained from measurements with the library images. A cylindrical lens, placed in front of the camera, improves the *z* resolution and accuracy. The streaming patterns are obtained using the DefocusTracker algorithm and the images are processed in 3D with the MATLAB program DefocusTracker [30,31].

The measured volume is divided into voxels with regular boxes of size *a*_*x*_ = 120 µm, *a*_*y*_ = 53 µm, and *a*_*z*_ = 60 µm. When a particle passes through a voxel, its velocity is calculated using the time and distance difference from its previous observation point by means of the nearest-neighbor algorithm. Each voxel holds the average velocity of all particles that have passed through it. The voxels are calculated such that they overlap by 75% in each dimension. The plotted velocity fields constitute slices through the volume, with the color indicating the two-dimensional (2D) velocity magnitude and the arrows indicating the velocity components in the cut plane. The slice thickness indicates the voxel size in the direction normal to the slice.

#### 7 Experimental procedure

The measurement procedure was as follows. The channel was flushed with a sequence of ethanol and water to avoid air bubbles at the walls and afterward filled with the sample solution containing water, dye, and tracer particles. The flow was stopped using the stop-flow valve connected to the outlet tube. The frequency of the sound was adjusted at the beginning of each experiment, without laser heating, while observing that the acoustically induced motion of suspended microparticles brings the particles toward the pressure node at the center of the channel. The laser is not visible during experiments and to aim the laser at the node of the acoustic field, the thermoacoustic streaming field is observed while adjusting the *y* coordinate of the point of incidence of the laser. When the field is symmetric, the laser is considered to be centered with respect to the acoustic field. In the thermoacoustic experiments, within a few seconds, the laser and then the acoustic field were turned on, and after reaching steady state, the acquisition started. In each acquisition, 1000 frames were recorded and each experiment was repeated 20 times, resulting in 18 814 (off-centered-laser) and 28 224 (centered-laser) particle trajectories. After each recording, the stop valve was opened and new sample solution was injected to avoid photobleaching of the dye and to ensure a random distribution of the particles in the channel.

To change the laser point of incidence to the side of the channel (for case I), the channel was moved along the *y* direction using a manual *x*-*y* stage. For the off-centered (asymmetrical) positioning of the laser, the actuation frequency was centered at 1.934 MHz with a sweep of 50 kHz and a sweep time of 1 ms. The experiments with varying dye concentrations (case II), including the centered (symmetric) position (case I), were carried out in a slightly different position in the channel (due to remounting of the chip) at which the resonance of the acoustic field was ideal, at 1.9712 MHz without sweep.

To assess the velocity field for no added dye, ten repeated measurements, of 500 frames each, were recorded. This procedure was also carried out for the purpose of validation of the quality of the acoustic field prior to each experiment.

#### 8 Numerical procedure

**Fig. 4:**
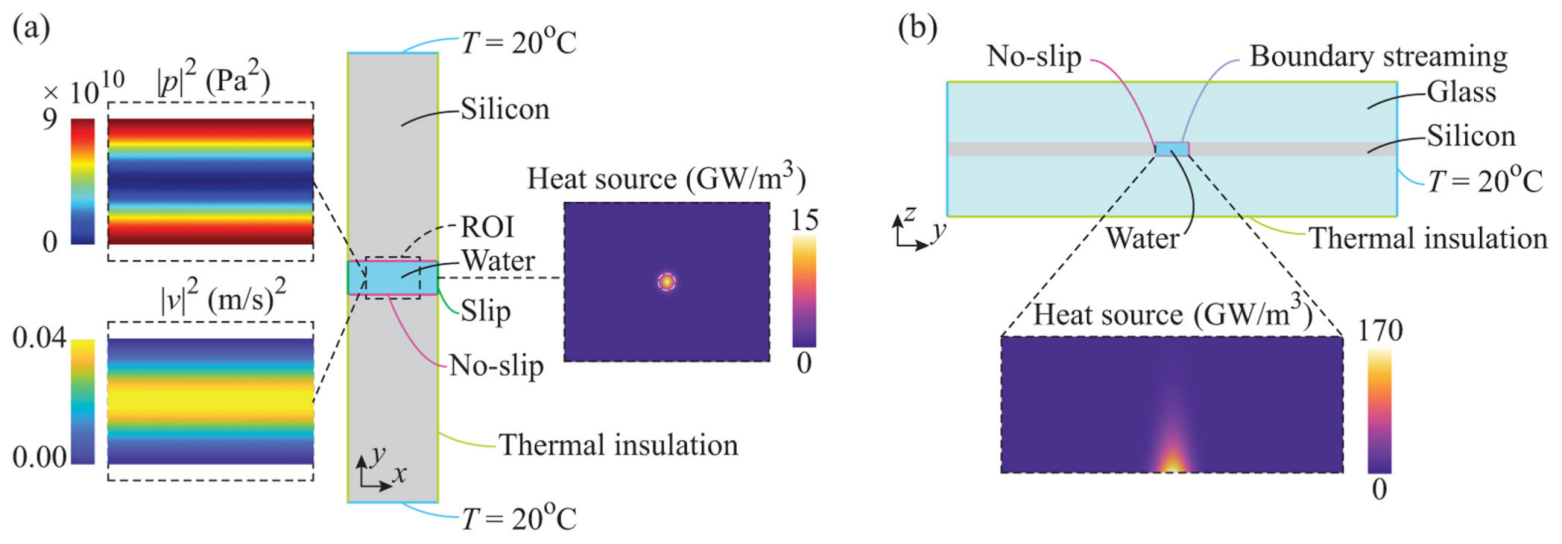
The simulation details indicating the boundary conditions in the model, showing (a) the simulated domain as viewed from the top, with the squared pressure and velocity fields, and the heat generated by the laser evaluated at *z* = *H/2*. (b) The cross-section view, with the laser heating evaluated at *x* = 0.

An effective numerical model was implemented in COMSOL Multiphysics v. 6.1 (COMSOL AB, Sweden). A 1-mm-long section of the chip, centered at the point of incidence of the laser, was modeled in three dimensions by means of the modules HEAT TRANSFER IN FLUIDS and LAMINAR FLOW using the built-in material properties for water, silicon, and borosilicate glass [32]. All other geometries are described in Sec. II B 2. The boundary conditions and the input fields are shown in Fig. 4. The laser light was modeled as a Gaussian beam of 1*/e*^2^ radius *ω*_0_ = 25 µm, with a total power of 5.8 mW, and was limited to the fluid domain comprising the built-in parameters for water. The laser heats the fluid by optical absorption according to Eq. (2), such that the heat is maximal near the channel floor and decays exponentially for increasing *z*. Outside the fluid domain, the solid materials were modeled using the built-in material parameters for silicon and borosilicate, respectively. A constant temperature of 293 K was assigned to the outer boundaries of the chip and thermal isolation was assigned to the *y*-*z* cross sections that limit the modeled volume. The model was fully coupled, so that the temperature field is included when solving the flow field and vice versa, although the streaming field is not expected to distort the temperature field for velocities below approximately 750 µm*/*s. The resulting thermal field was used as model input for the flow simulation in the fluid domain. Symmetry boundary conditions were applied such that only half a channel was modeled for case I and a quarter of the channel was modeled for case II due to the symmetrical positioning of the laser.

A plane standing wave along the *y* direction of 10 J*/*m^3^ acoustic energy density was assigned to the fluid domain with maximum pressure at the walls and maximum velocity in the center of the channel. The acoustic body force was applied in all dimensions by taking the gradients of the temperature-dependent density and compressibility fields and inserting them in Eq. (3). The effect of gravity was included. The steady background flow from Rayleigh streaming was implemented by a no-slip sliding wall of velocity $u_{\text{str }}(y)=\left(3v_{\text{A}}^{2}/8c_{0}\right)\text{sin}\left(2k_{y}y\right)$, assigned to the channel floor and ceiling [16], where *c*_0_ is the speed of sound in water at ambient temperature and *k*_*y*_ is the wave number. The channel side walls were set to be no-slip, whereas the *y*-*z* surfaces at the end of the modeled volume were set to a slip boundary condition.

The plotted simulated velocity fields constitute infinitesimally thin slices through the volume, with the color indicating the 2D streaming velocity magnitude and the arrows indicating the streaming velocity components in the cut plane. Since the temperature field was not measured experimentally, comparisons between the simulated and experimental streaming fields are only qualitative. Further details of the numerical procedure are given in the Appendix.

## III Results And Discussion

To investigate how spatially differing thermal gradients affect the thermoacoustic streaming field, we conducted two experiments. First, we investigated the effect of two different locations of the laser heat source in the width dimension of the channel (*y*). Second, we observed the effect of modulating the light absorption in the fluid by varying the concentration of the near-infrared-absorbing ICG dye.

### A Case I: Varying the laser positioning and thermal gradient

**Fig. 5:**
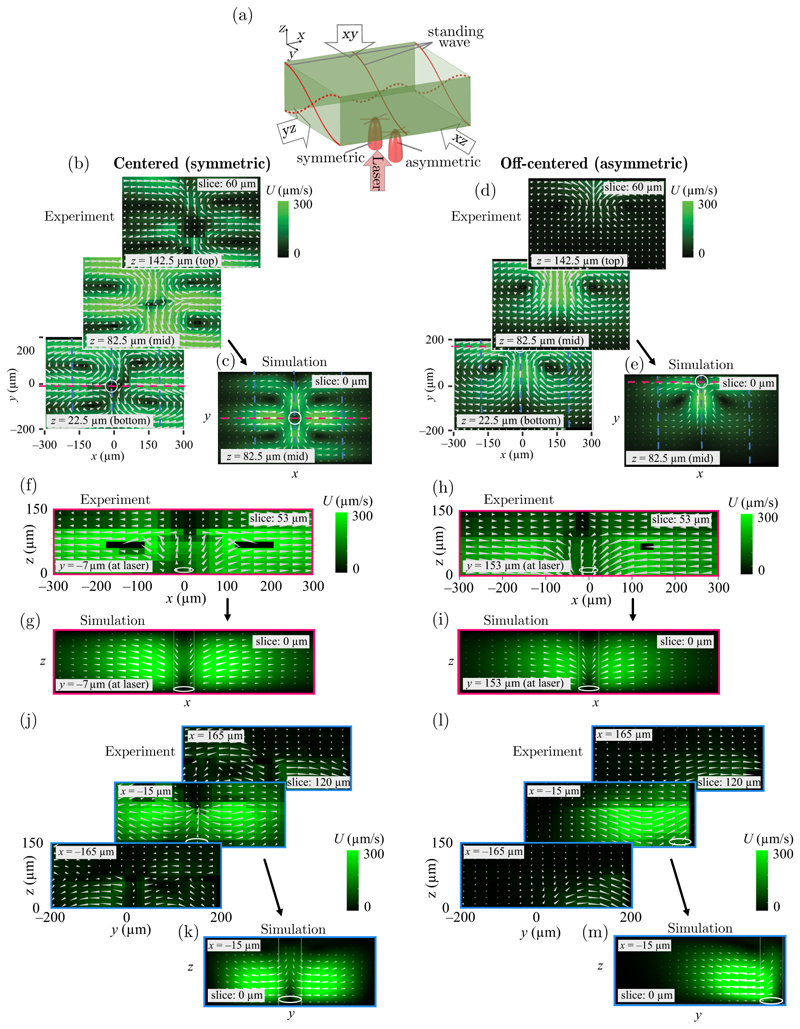
Varying the laser location (case I). (a) The orientation of the channel, the laser, and the standing-wave field. (b)–(m) The in-plane velocity magnitude (color) and its components (arrows) for different projections and laser-beam positions (white circles). The location and thickness of each analyzed slice volume is indicated in the text box on each slice. The dashed magenta and blue lines indicate the locations of the cut planes. (b),(c) Top-view projections of the streaming fields in (b) experiment and (c) simulation for the centered laser. (d),(e) Top-view projections of the streaming fields in (d) experiment and (e) simulation for the off-centered laser. (f),(g) Side-view projections of the streaming fields in (f) experiment and (g) simulation for the centered laser. (h),(i) Side-view projections of the streaming fields in (h) experiment and (i) simulation for the off-centered laser. (j),(k) Cross-section-view projections of the streaming fields in (j) experiment and (k) simulation for the centered laser. (l),(m) Cross-section-view projections of the streaming fields in (l) experiment and (m) simulation for the off-centered laser.

The point of incidence of the laser will affect the location of the thermal gradient within the channel. The shape of the streaming is due to the acoustic-body-force field, which is influenced by the temperature gradient. The body force field is a result of the interaction between the acoustic pressure and the compressibility field. To summarize, the acoustic pressure and compressibility field create an acoustic-body-force field, which in combination with the temperature distribution drives the thermoacoustic streaming. Thus, the change in position of the heat source in the acoustic field results in a change in the thermoacoustic streaming pattern. We investigated this effect by changing the location of the laser spot relative to the acoustic field by positioning it in the channel center versus near the side wall [see Fig. 5(a)]. In Video 1, we show the evolution of the thermoacoustic streaming field as the laser point of incidence is slowly moved across the width of the channel. As indicated in Fig. 1(d), the resulting acoustic body force landscape leads to streaming patterns that we can observe experimentally by tracking the suspended fluorescent microparticles evaluated in a 3D section.

In Fig. 5(a), we show a graphic illustrating the perspective of view. In Figs. 5(b)–5(e), we show the top view (*x*-*y* plane) of the thermoacoustic streaming at different *z* positions, along the optical axis of the laser beam, for symmetric and asymmetric laser positioning. By visualizing the measured velocity field as viewed along the direction of the laser light, we observe four streaming rolls for the centrally positioned heat source [Figs. 5(b) and 5(c)] and two rolls for the off-centered positioning [Figs. 5(d) and 5(e)].

The minimum velocity is found in the center of each streaming roll and increases to approximately 300 µm*/*s where two rolls flow in parallel. In the centered case, as shown in Fig. 5(b), the velocity also drops in the center of the channel, where all rolls meet. The underlying flow is driven by the acoustic body force induced by the temperature gradient around the laser spot and the pressure field, which is highest near the walls. Therefore, in the centered case, the liquid is pushed in from both sides, transporting warm liquid out of its way. Because thermal diffusion is faster than thermoacoustic streaming in this case, the escaping fluid quickly cools down when it leaves the heated region, since thermal diffusion dominates over advection. In the off-centered case, the driving mechanism is caused by an acoustic body force that acts along the *x* direction, transporting liquid from both sides toward the heated region. Hence, the fluid escapes along the *y* direction, where the opposing body force is weaker. For both the centered and off-centered laser-spot positions, the velocity is lowest at the top and bottom of the channel and high between the midheight and bottom of the channel. The corresponding simulations are in good agreement with the experimental observations [see Figs. 5(b) and 5(e)].

The *x*-*z* slices in Figs. 5(f)–5(i) are located at the point of incidence of the centered (*y* = −6.6 µm) and off-centered (*y* = 153 µm) laser positions, respectively. In the centered case, we can observe a motion in the *z* direction, away from the point of incidence of the laser [see Figs. 5(f) and 5(g)]. On the contrary, in the off-centered case, the flow is directed toward the laser both in experiments and in simulations [see Figs. 5(h) and 5(i)]. This effect is related to the highly absorbing fluid (99% of the incoming light) and thus the temperature field has a strong *z* dependence. Consequently, the body force is directed from all directions toward a point just above the channel floor. However, in the center of the channel, the magnitude of the downward force is weak due to the zero-pressure condition. Instead, fluid is pushed in from the sides at the lower half of the channel, resulting in a net upward flow for the centrally located laser [see Figs. 5(f) and 5(g)]. For the side-located laser [see Figs. 5(h) and 5(i)], the pressure amplitude at that channel position is higher and therefore there is a considerable downward component of the body force. As a result, the fluid instead flows downward and escapes along the decaying pressure gradient in the *y* direction, toward the center of the channel.

The *y*-*z* slices in Figs. 5(j)–5(m) show the cross-section views along the *x* direction with one slice located at the point of incidence of the laser (*x* = −15 µm and one on each side, at *x* = −16 µm and *x* = 165 µm), for both the centered laser and the off-centered laser-spot positions. For the centered laser [see Figs. 5(j) and 5(k)], the middle cut plane (at *x* = −15 µm) shows that liquid moves toward the heat source with a minor *z* component, leading to curved stream lines. The velocity drops near the center where the flow becomes directed out of the plane. For the off-centered laser position [see Figs. 5(l) and 5(m)], the middle cut plane (at *x* = −15 µm) shows that the flow is highest near the channel floor. The reason for this flow is a substantial downward force, resulting from the high temperature at the point of incidence of the laser light compared to a lower temperature at the ceiling.

### B Case II: Effect of varying the laser absorptivity of the medium

We investigated how an increase in laser light absorption in the medium affects the thermoacoustic streaming field. This was achieved by varying the concentration of ICG dye in the fluid for a laser beam located in the center of the channel. Low absorption allows light to penetrate through the entire height of the channel, leading to near-uniform heating along the *z* direction. A higher concentration of ICG causes more light to be absorbed close to the point of incidence of the laser beam. Therefore, it was expected that the increase in absorption would lead to higher overall thermoacoustic streaming velocities and a more complex streaming pattern in the *y*-*z* cross section due to the more pronounced *z* dependence of the thermal field.

**Fig. 6:**
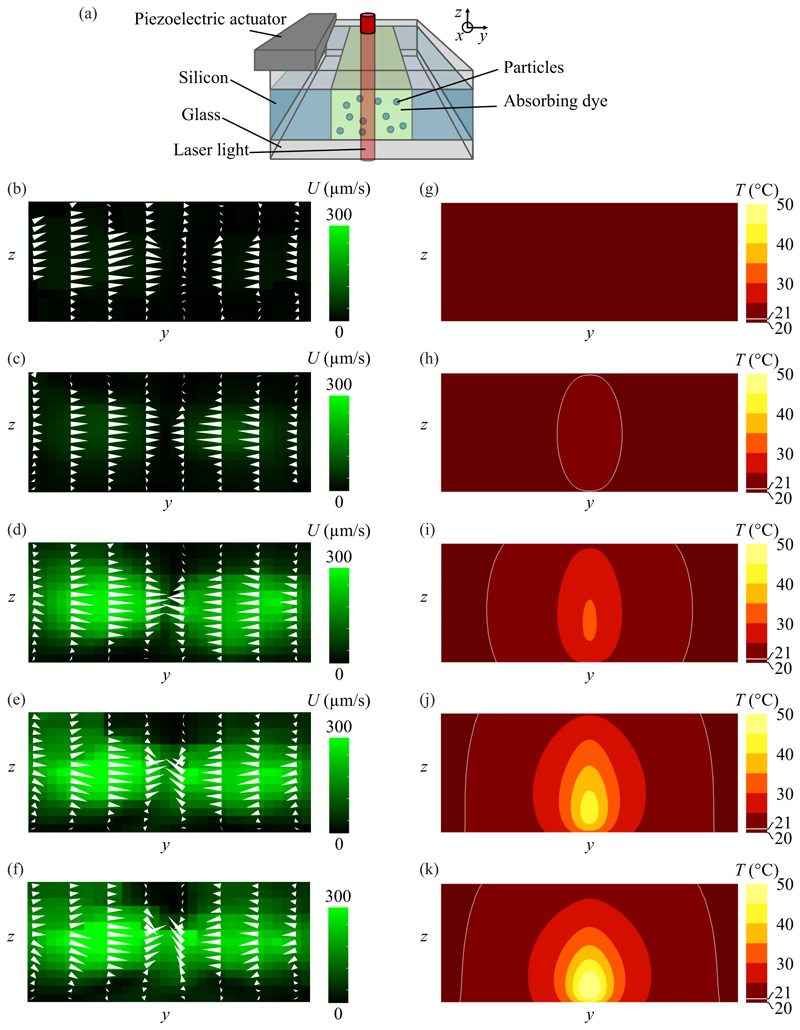
The thermoacoustic streaming when increasing the absorption in the medium. (a) The configuration of the laser illumination. (b)–(k) The (b)–(f) *y*-*z* cross-section views of the measured streaming field for an absorbed fraction ranging from 0% to 99% (the same data set as in Fig. 5) and (g)–(k) the corresponding simulated temperature fields. Absorbed fraction: (b),(g) 0%; (c),(h) 10%; (d),(i) 50%; (e),(j) 90%; (f),(k) 99%. The velocity magnitude (color) and velocity components (arrows) correspond to a slice thickness of 120 µm around the point of incidence of the laser.

In Fig. 6, we show the generated thermoacoustic streaming velocity field in the *y*-*z* cross section of the channel for a centered laser spot and an absorbed fraction of light ranging from 0 to 99%. In general, the thermoacoustic velocity increases with increasing absorption in the medium. For no dye added, there is no temperature gradient and we observe the background velocity field of the polystyrene particles due to the acoustic radiation force and the boundary-driven acoustic streaming. The polystyrene tracer particles are 1.9 µm in diameter and thus close to the critical radius at which the radiation force and acoustic streaming are similar in magnitude [33]. Hence, the four characteristic streaming rolls become smaller [see Fig. 6(b)], as described in Ref. [33]. For 10% absorbed light, the thermoacoustic streaming is found with no *z* component. This is because the laser heat source extends through the entire height of the channel and causes a temperature field that is almost invariant along the *z* direction. Thus, the thermoacoustic body force will act primarily in the *x*-*y* plane. For higher absorptions, the flow fields diverge in the *z* direction near the walls and then converge when approaching the center of the channel [see Figs. 6(d)–6(f)].

The observed thermoacoustic streaming in the *y*-*z* cross section is different from the observations made by Qiu *et al*. [13], which were conducted for 99% absorption. In that work, two full streaming rolls formed in the *y*-*z* cross section. The reason lies in the different temperature gradients induced by the laser light in this work and by a high-power LED in Ref. [13], which, in turn, affect the streaming pattern and the velocity field. It was found that the thermoacoustic streaming has a predominantly *y* component in the *y*-*z* cross-section plane [see Fig. 6(f)], when the laser spot is centered in the channel with an absorbed fraction of 99%. As a result, no distinct streaming rolls are observed in the *y*-*z* plane, which contrasts with the behavior when an LED is used as the heating source with the same absorbed fraction. This suggests that, in the case of laser heating, the fluid is pushed by the acoustic body force toward the channel center but primarily escapes along the *x* direction. In contrast, under LED heating, the fluid escapes in both the *x* and *z* directions. This difference can be attributed to the distinct distributions of the acoustic body force in the two cases. While the acoustic body force is dominant along the *y* direction in both scenarios, the amplitude of the acoustic body force induced by laser heating is smaller than that induced by LED heating, for a given pressure amplitude, despite the fact that the temperature gradient along the *y* direction is much steeper in the laser-heating case (160 K/mm, versus 10 K/mm for LED heating). This is because the temperature gradient in laser heating is concentrated near the channel center (the low-pressure region) due to the small size of the laser spot, whereas the temperature gradient is more broadly distributed along the *y* direction with LED heating. The downward force due to the temperature gradient along *z* is similar in both cases, but relative to the *y* component, the laser heating has a more dominant *z* component which prevents roll formation in the *y*-*z* cross section in the case of the laser heating and thus fluid escapes only along *x*. Further decreasing the laser-spot size beyond the size investigated herein is not expected to lead to considerably more confined flow rolls in the steady regime, since the same amount of thermal energy is deposited and the resulting temperature field would be essentially the same due to the fast thermal diffusion.

## IV Conclusions And Further Studies

A setup was built to generate laser-induced temperature gradients in an acoustically actuated microchannel. Through experiments and numerical simulations, we have shown that moving the heat source away from the center results in a change in the thermoacoustic streaming pattern. Increasing the light absorption of the medium leads to faster thermoacoustic streaming. This has relevance when applying this technology to relocate the fluid orthogonally to the channel. Then, a high transmission is desirable and the lower velocity must be compensated for by increasing the acoustic field amplitude or the laser power. The simulated temperature fields indicate that for the employed laser power, temperatures may reach levels above 37°C when the medium is highly absorbing. For potential applications involving living cells, viability tests both regarding temperature effects and toxicity of the medium will be essential. To achieve fast thermoacoustic streaming at lower temperatures, the acoustic field amplitude can instead be increased, as suggested by Eqs. (3) and (4). In future works, we plan to investigate the transient behavior of the fluid during the onset and decay of the temperature gradient. Additionally, temperature measurements will be conducted to verify the simulation results. Deng *et al*. [34] have used lifetime imaging of temperature sensitive particles to map the temperature in their acoustofluidic systems. A similar approach is an option for future experiments on the presented setup. We believe that this work can lay the ground for schemes to tailor more advanced spatiotemporal streaming flows inside microfluidic channels, with potential applications in the field of cell separation and sorting.

## Supplementary Material

Video 1

Supplementary Materials

## Figures and Tables

**Video 1:**
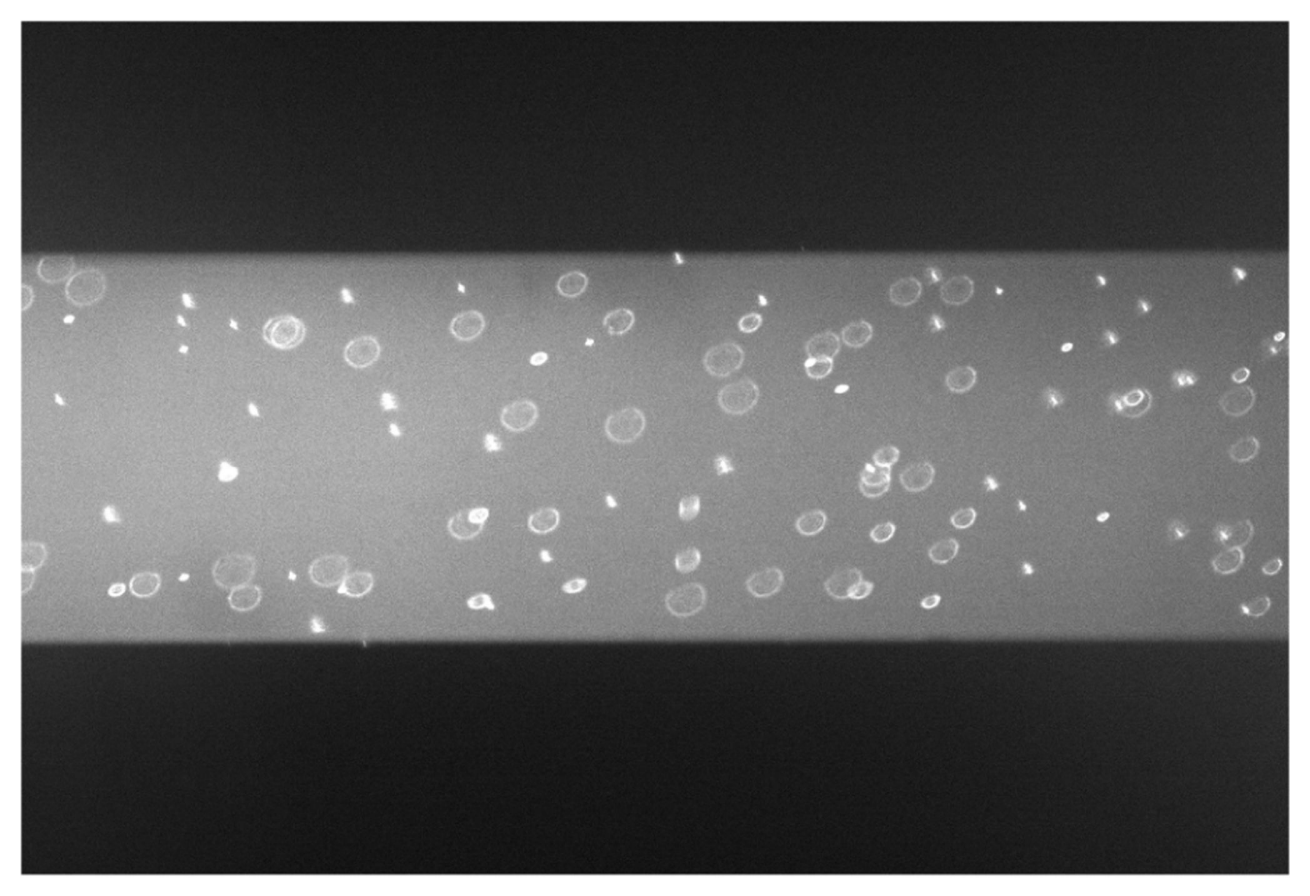
A real-time recording of the thermoacoustic streaming of 1-μm-diameter fluorescence tracer particles for a sound field generated at 1.9 MHz and for an applied amplitude of 2 V. The laser power was set to 10 mW and the laser point of incidence was continuously moved along y (top to bottom in the video) by manually adjusting the stage. In total, 380 frames were recorded at 20 frames/s.

**Table I:** The different ICG dye concentrations and the corresponding transmission for case II.

*c* (mg/ml)	Transmission (%)	Light absorbed (%)
0	100	0
0.00014	90	10
0.00074	50	50
0.00238	10	90
0.00473	1	99

**Table II:** The boundary conditions.

Boundary type	Equation
Thermal isolation	**−*n* · *q*** = 0, with normal vector ***n***
Temperature	*T* = 293 K
No slip	***u*** = 0
Slip	***u*** · ***n*** = 0
Boundary streaming	*u_y_ = u*_str_ *(y)*
Pressure constraint point	*p* = 0 Pa (assign anywhere in fluid)
