# Supplementary material for: Configurable thermoacoustic streaming by laser-induced temperature gradients
Source: Phys Rev Appl. Author manuscript; Available in PMC 2025 Sep 25. (PMC7618173; doi:10.1103/physrevapplied.23.024043)
Supplement: Supplementary Materials [file EMS208791-supplement-Supplementary_Materials.pdf]

APPENDIX: SIMULATION DETAILS

The stationary temperature and acoustic fields were computed in COMSOL by the fully coupled physics interfaces LAMINAR FLOW and HEAT TRANSFER IN FLUIDS with solid domains. The temperature field T was solved using the following equation:

$$\rho c_p \boldsymbol{u} \cdot \nabla T + \nabla \cdot \boldsymbol{q} = Q, \tag{A1a}$$

$$\boldsymbol{q} = -k_{\mathrm{th}} \nabla T, \tag{A1b}$$

TABLE II. The boundary conditions.

Boundary type	Equation
Thermal isolation	$-\boldsymbol{n} \cdot \boldsymbol{q} = 0$, with normal vector $\boldsymbol{n}$
Temperature	$T = 293$ K
No slip	$\boldsymbol{u} = 0$
Slip	$\boldsymbol{u} \cdot \boldsymbol{n} = 0$
Boundary streaming	$u_y = u_{\mathrm{str}}(y)$
Pressure constraint point	$p = 0$ Pa (assign anywhere in fluid)

where ρ is the density, c_p is the specific heat capacity, $\boldsymbol{u}$ is the velocity field, $\boldsymbol{q}$ is the heat flux, Q is the heat source, and k_{th} is the thermal conductivity.

The thermoacoustic streaming field $\boldsymbol{u}$ was solved using the following equations:

$$\rho(\boldsymbol{u} \cdot \nabla)\boldsymbol{u} = \nabla \cdot [-p\boldsymbol{I} + \boldsymbol{K}] + \boldsymbol{f}_{\mathrm{ac}} + \rho \boldsymbol{g}, \tag{A2a}$$

$$\rho \nabla \cdot \boldsymbol{u} = 0, \tag{A2b}$$

$$\boldsymbol{K} = \eta(\nabla \boldsymbol{u} + (\nabla \boldsymbol{u})^{\top}). \tag{A2c}$$

The boundary conditions are shown in Table II.

The laser heat source was modeled as

$$Q = P_{\mathrm{laser}} \cdot 2/(\pi \omega_0^2) \cdot a \cdot \mathrm{gauss2D}(x, y) \cdot \exp(-az), \tag{A3a}$$

$$\mathrm{gauss2D}(x, y) = \exp(-2 \cdot [(x - x_{\mathrm{laser}})^2 + (y - y_{\mathrm{laser}})^2]/\omega_0^2), \tag{A3b}$$

with optical power P_{laser} and attenuation coefficient $a = -\log(\mathrm{Tr})/H$, where H is the height of the channel. The mesh was a free tetrahedral mesh with a maximum element size of 15.9 μm in the fluid domain and 50 μm in the solid domains, as validated via mesh convergence to within 5% compared to the finest mesh by probing the thermoacoustic streaming velocity field across the y direction.
